# The Potential Role of Large Language Models in Assisting Patients and Guiding Emergency Care Visits

**DOI:** 10.3390/jcm15083170

**Published:** 2026-04-21

**Authors:** Kristina Gerhardinger, Josina Straub, Julia Lenz, Siegmund Lang, Volker Alt, Borys Frankewycz, Maximilian Kerschbaum, Lisa Klute

**Affiliations:** 1 Department of Trauma Surgery, University Hospital Regensburg, Franz-Josef-Strauss-Allee 11, 93053 Regensburg, Germany; 2 Emergency Department, University Hospital Regensburg, Franz-Josef-Strauss-Allee 11, 93053 Regensburg, Germany

**Keywords:** large language models, emergency care, patient triage, artificial intelligence, health communication

## Abstract

**Background/Objectives**: Overcrowding in emergency departments (EDs) remains a critical challenge in modern healthcare systems, driven in part by patient uncertainty regarding symptom urgency and a lack of accessible medical guidance. Recent advances in artificial intelligence, particularly large language models (LLMs), present a novel opportunity to support patient navigation and relieve pressure on ED infrastructures. **Methods**: A total of 238 unique patient questions were identified through a structured web search. Following deduplication and thematic clustering, 15 representative questions were selected. Each question was submitted to the three LLMs—ChatGPT (v3.5), DeepSeek, and Gemini—using a standardized prompt. Responses were assessed by clinical experts (N = 8) who were blinded to the model source. Reviewers selected the best overall response per question, as well as the individual responses of the three LLMs for each respective question. **Results**: ChatGPT was selected as the best-performing model in 60% of cases, with DeepSeek and Gemini selected in 23% and 17%, respectively. ChatGPT responses also achieved the highest proportion of “excellent” quality ratings and the lowest proportion of “unsatisfactory” outputs. Across all models, clarity was the most positively rated domain (79% agreement), followed by empathy (72%), length/detail appropriateness (71%), and completeness (65%). Over two-thirds of raters expressed willingness to integrate LLM-based tools into clinical practice for patient education and pre-triage counseling. **Conclusions**: Large language models demonstrate promising capabilities in responding to emergency care-related patient queries. Their ability to deliver medically sound and communicatively effective answers positions them as potential digital adjuncts in the management of low-acuity ED presentations and prehospital triage.

## 1. Introduction

Western healthcare systems are increasingly confronted with structural and operational limitations that compromise time-efficient and appropriate patient care. One of the most visible consequences of this imbalance is the persistent overcrowding of emergency departments (EDs), a phenomenon driven not solely by the incidence of acute conditions, but by broader system-level deficits [1]. Emergency department overcrowding has been a known issue for decades [1]. In particular, the inability of general practitioners and specialist practices to accommodate patients within clinically acceptable timeframes often leads individuals with non-emergent symptoms to seek care in EDs. This misallocation of healthcare resources impedes efficient triage, prolongs waiting times, and exerts substantial pressure on clinical staff [2,3,4].

Compounding this issue is the frequent lack of accessible, evidence-based guidance for patients attempting to determine the urgency of their symptoms. In the absence of timely consultation options, many patients default to emergency services out of an abundance of caution. This pattern reflects a systemic failure to align patient behavior with appropriate levels of care, and it contributes significantly to the overutilization of emergency infrastructure.

Concurrently, recent advances in artificial intelligence (AI)—and more specifically in neural networks, deep learning algorithms, and large language models (LLMs)—have generated increasing interest in their potential applicability within clinical medicine [5]. While sectors such as finance, logistics, and legal services have rapidly adopted AI technologies, healthcare has been comparatively conservative in implementation. Nonetheless, the current generation of LLMs demonstrates capabilities that extend beyond simple information retrieval, including natural language processing, context-aware dialogue generation, and synthesis of complex clinical content [6].

Hitherto, the use of LLMs in clinical environments remains largely exploratory [7]. Most applications are limited to non-interactive, question-answering tasks and do not yet leverage the full potential of these systems for dynamic, context-sensitive patient engagement or clinical decision support. However, emerging literature suggests that LLMs may assist in a range of functions, including the stratification of patient risk, optimization of documentation workflows, and facilitation of patient-provider communication.

Given the persistent resource constraints within healthcare delivery and the rising burden on emergency services, the potential role of LLMs in improving patient navigation, triage accuracy and system efficiency warrants systematic investigation [2,8,9,10].

## 2. Materials and Methods

This prospective exploratory study evaluated the ability of large language models (LLMs) to support patient decision-making regarding the necessity of emergency department (ED) visits. The study focused on how accurately, clearly, and empathetically three state-of-the-art LLMs—ChatGPT (v3.5), DeepSeek (R1), and Gemini (2.0 Pro)—responded to common patient questions related to emergency care. The study design can be understood as a structured evaluation framework for assessing LLM performance in pre-triage contexts, comprising four key components: (1) systematic scenario selection, (2) standardized prompting, (3) blinded expert evaluation, and (4) multidimensional quality assessment. This framework is designed to be transferable across different model generations and clinical contexts.

### 2.1. Question Identification and Selection

An initial comprehensive Google search was conducted using the query: “frequently asked questions AND emergency department OR when do I need to go to the hospital?” This search returned approximately 245,000,000 results within 0.46 s (conducted on 3 February 2025; region setting: Germany). The first 100 websites were systematically screened by two independent reviewers. Pages were included if they (1) were published after 1 January 2017, (2) were available in English or German, and (3) contained structured FAQ or Q&A formats targeting patients. Exclusion criteria included provider- or product-specific content, institution-bound triage recommendations, or non-medical/administrative information (see Table 1).

From this screening, 238 unique patient questions were identified. After deduplication and thematic clustering, 25 questions were classified as redundant. A final list of 15 representative and semantically diverse questions was curated to reflect the most commonly asked patient concerns regarding emergency care (see Table 2 and Table 3).

### 2.2. LLM Interaction and Prompting

Each of the 15 questions was submitted individually to ChatGPT (OpenAI), Gemini (Google), and DeepSeek (DeepSeek AI), accessed in March 2025 via their publicly available interfaces. To ensure standardization and minimize contextual bias, each interaction was initiated in a new chat window. All models received the same instruction prompt:

“You are an experienced emergency physician. Please provide a concise, clear, and empathetic answer to the following patient question. Your response should be understandable to laypersons, limited to 150 words, and include relevant clinical warning signs.”

Each model produced one response per question, resulting in 45 total responses (15 questions × 3 models).

### 2.3. Evaluation Process

The responses were anonymized, randomized, and presented to a panel of healthcare professionals with experience in emergency and acute care. For each question, raters were asked to:

1. Select the best overall response from three LLM-generated answers.
2. Evaluate the individual responses of the three LLMs for each respective question (4-point scale: excellent—no clarification necessary/satisfactory with minimal clarification necessary/satisfactory with moderate clarification necessary/unsatisfactory—major clarification necessary). If a response received a negative evaluation, the raters could indicate predefined reasons for the critique (e.g., “off-topic,” “factual errors,” “excessive or insufficient detail,” “unclear language”).
3. Evaluate the total content of all responses for each question regarding completeness of content, empathy and professionalism, clarity and understandability and length and detail appropriateness (5-point scale: I strongly agree/I agree/neutral/I do not agree/I strongly do not agree) (see Table 4).

### 2.4. Exploratory Feedback

After rating all answers, participants completed a set of exploratory questions assessing their overall impressions of AI-generated responses. This included attitudes toward:

- The utility of LLMs in real-world clinical workflows,
- Potential to reduce clinician workload in the ED,
- Appropriateness of responses for patients without medical training,
- Willingness to integrate AI-based tools in practice.

### 2.5. Statistical Analysis

Descriptive statistics were reported as absolute values, percentages, means, and standard deviations. The frequency with which each LLM’s response was selected as “best” was calculated for each question and in aggregate. All statistical analyses were performed with IBM SPSS Statistics version 28.0 and Microsoft Excel version 2302. Inter-rater reliability for Likert-scale evaluations was assessed using the intraclass correlation coefficient (ICC).

As the study involved no patient data and only public, AI-generated content, ethics board review was not required.

## 3. Results

### 3.1. Overall Performance Across Large Language Models

**Figure 1:**
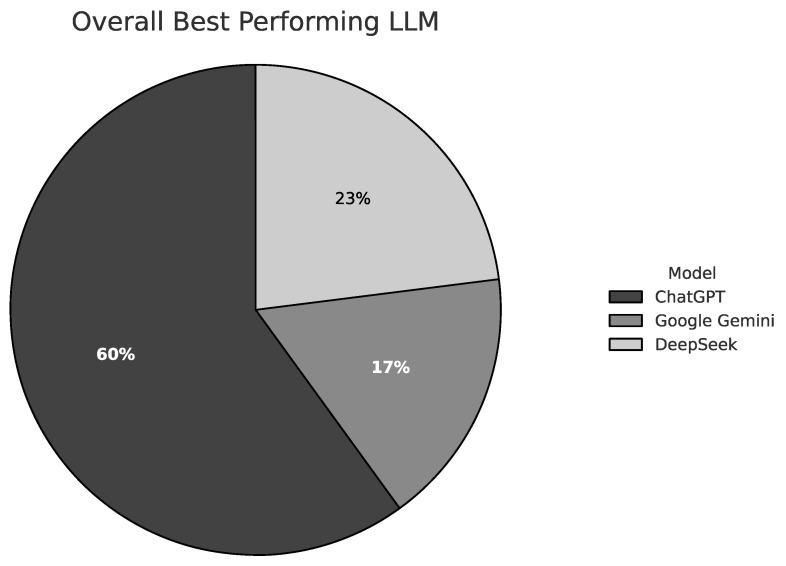
Pie chart: Total percentage of “Best Overall LLM.”.

Among the 15 standardized emergency-related scenarios, ChatGPT was most frequently selected as providing the best overall response. Over all questions, it was preferred in 60% of cases, while DeepSeek and Google Gemini were selected in 23% and 17%, respectively (Figure 1). This result underscores ChatGPT’s general dominance in both symptom-based and diagnosis-oriented prompts.

### 3.2. Response Quality Ratings

**Figure 2:**
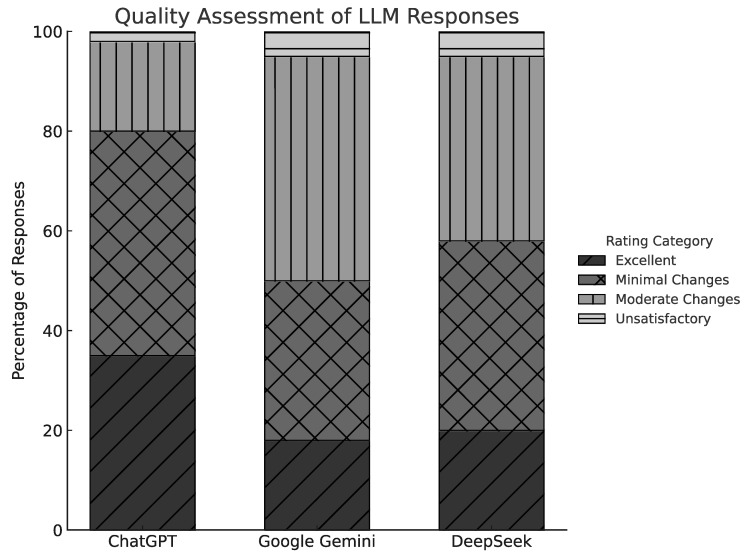
Quality assessment ratings by model.

Each AI-generated answer (N = 45) was rated using a 4-level classification: excellent, satisfactory with minimal clarification, satisfactory with moderate clarification, and unsatisfactory. As shown in Figure 2, ChatGPT achieved the highest percentage of “excellent” ratings (35%) and the lowest rate of “unsatisfactory” responses (2%). DeepSeek and Gemini, while both generally adequate, had higher proportions of responses rated as requiring moderate clarification (37% and 45%, respectively), and slightly more “unsatisfactory” judgments (5% each). These findings suggest that ChatGPT’s answers were perceived as more reliable and immediately usable, while responses from the other models often required editorial or factual refinement before being suitable for patient communication.

**Figure 3:**
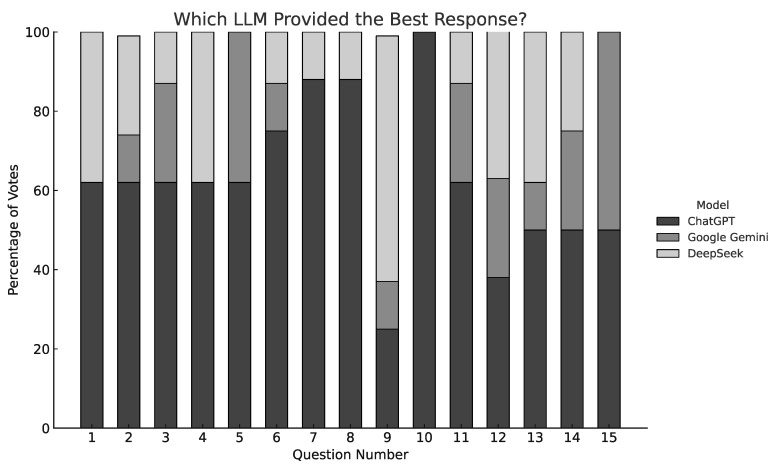
Percentage of “Best Answer” selections across 15 questions (ChatGPT, Gemini, DeepSeek).

A per-question breakdown (Figure 3) confirmed this trend: ChatGPT was rated highest in 11 out of 15 items. In specific scenarios—such as blurred vision, pediatric fever, and medication overdose—Gemini and DeepSeek occasionally surpassed ChatGPT, suggesting that some individual model strengths may be context-dependent.

### 3.3. Evaluation Across Qualitative Dimensions

Beyond categorical quality ratings, the total content of all responses for each question was evaluated along four qualitative axes using a 5-point Likert scale (see Table 4). Completeness of content, empathy and professionalism, clarity and understandability, and length/detail appropriateness were assessed.

**Figure 4:**
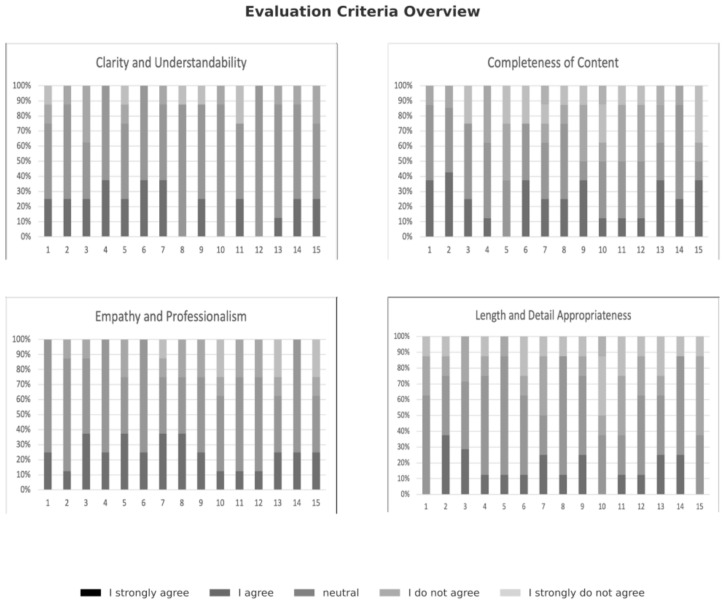
Four-panel chart: Completeness of Content, Empathy and Professionalism, Clarity and Understandability and Length and Detail Appropriateness.

As illustrated in Figure 4, the evaluations vary markedly across all four criteria depending on the specific question. In the domain of “completeness of content”, responses to questions 1, 2, and 14 were rated particularly positively. In addition, for “empathy and professionalism”, four questions received responses exclusively in the categories “I strongly agree” or “I agree.” Regarding “clarity and understandability” and “Length and Detail Appropriateness”, the responses to the 15 individual questions displayed considerable variability.

The dimensional evaluation highlights the multifaceted nature of quality in AI-generated content: While factual completeness is essential, communication style and perceived professionalism play a central role in overall reception.

### 3.4. Summary Agreement Across Dimensions

**Figure 5:**
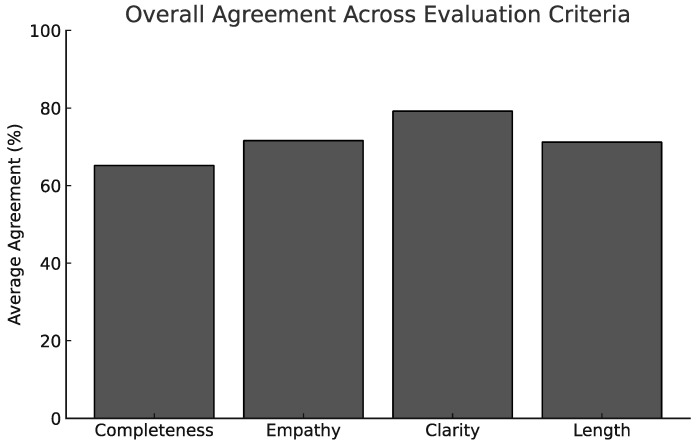
Summary bar chart showing average agreement per evaluation criterion.

As summarized in Figure 5, overall agreement with the statements regarding quality was high.

- Clarity and Understandability emerged as the most positively rated domain overall, with 79% of answers rated as “agree” or “strongly agree”.
- Empathy and Professionalism followed closely with 72% positive agreement, reflecting the perceived patient-centered tone of the LLM outputs.
- Length and Detail Appropriateness received 71% approval, suggesting that most responses struck a good balance between brevity and completeness.
- Completeness of Content showed the widest spread, with only 65% positive agreement—likely influenced by LLM limitations in providing nuanced clinical differentials or guideline-based recommendations.

Mean approval (sum of “agree” and “strongly agree”) was above 70% for all four dimensions, emphasizing the general acceptability of LLM-generated responses for simulated clinical questions. These findings suggest that current AI models are not only capable of syntactically coherent answers but also of producing content that aligns well with the expectations of trained clinicians. Inter-rater reliability was low across all evaluated dimensions (Completeness: ICC = 0.021; Empathy/Professionalism: ICC = −0.175; Clarity/Understandability: ICC = −0.180; Length/Detail Appropriateness: ICC = 0.054), indicating substantial variability in expert ratings. Two domains (empathy/professionalism and clarity/understandability) yielded negative ICC values, indicating inter-rater agreement below chance level.

### 3.5. Expert Attitudes Toward Clinical Integration

In the final section of the evaluation, reviewers were asked whether they would support the integration of LLMs into real-world clinical workflows. All participants expressed a fundamentally positive attitude toward the use of AI for patient education, triage support, and initial guidance in emergency settings. Several raters explicitly stated that LLM-based assistants could help mitigate common system bottlenecks—such as overcrowded emergency departments and lack of access to general practitioners—by enabling patients to better assess urgency and seek appropriate levels of care.

Importantly, none of the reviewers viewed LLMs as replacements for physicians. Instead, they emphasized their value as a complementary tool for structured pre-visit communication and the dissemination of standardized information. While isolated concerns were raised regarding the risk of misinformation or patient overreliance on AI-generated answers, the consensus was that, with proper guardrails and human oversight, such tools could improve efficiency and reduce cognitive burden in frontline care.

## 4. Discussion

This study provides a comparative evaluation of three advanced large language models—ChatGPT, Google Gemini, and DeepSeek—in addressing frequently asked patient questions related to emergency care. The results demonstrate that LLMs, when prompted appropriately, can deliver responses that are not only medically relevant but also comprehensible and empathetically phrased. ChatGPT consistently outperformed the other two models across multiple domains, particularly with regard to clarity, completeness, and perceived overall quality. The increasing interest in LLMs for potential applications in emergency medicine further supports this direction [11]. The architecture of these LLMs, often based on transformer networks, allows for nuanced understanding and generation of text [12]. It’s worth noting that the timeline of mainstream LLMs commercially available to the public signifies major advancements in LLMs within the field of natural language processing [13].

The high proportion of “excellent” and “minimal change” ratings attributed to ChatGPT aligns with prior research indicating that transformer-based models are capable of generating accurate and patient-appropriate explanations across a range of medical domains. In our analysis, ChatGPT was selected as the best-performing model in 60% of all evaluated cases. Moreover, it received the fewest “unsatisfactory” ratings, supporting the model’s robustness in responding to clinical information needs posed in lay language. General purpose language models trained on clinical notes have demonstrated early potential as all-purpose prediction engines in healthcare [14]. Given that common ED complaints include a great uncertainty whether there is a need for urgent medical attention, the ability of LLMs to address these questions accurately is particularly valuable [2].

While ChatGPT exhibited strengths in clarity and communication, its performance in the domain of content completeness was more variable. Certain responses lacked critical nuance, particularly in questions addressing differential diagnoses or procedural indications—highlighting a well-described limitation of LLMs: the tendency toward surface-level generalizations in the absence of clinical context. In contrast, Google Gemini and DeepSeek showed broader variability across all assessed dimensions, with a higher incidence of ratings requiring moderate or substantial clarification. As LLMs are applied to clinical tasks, it is important to consider in what areas of medicine these models can be most impactful [15].

Importantly, the structured evaluation across four criteria—completeness, empathy, clarity, and length/detail appropriateness—revealed significant inter-item variation. The strongest agreement was observed in the dimension of clarity, where 79% of raters either agreed or strongly agreed that responses were understandable and appropriately phrased. Conversely, the lowest agreement was observed for completeness of content, suggesting a need for future optimization of LLM prompts or post-processing algorithms to ensure the inclusion of clinically relevant details. The reported findings represent aggregated patterns of expert judgment across all evaluated responses and should be interpreted accordingly, rather than as discrete per-item agreement measures.

The results also underscore a noteworthy openness among clinical evaluators toward integrating LLMs into routine medical workflows. More than two-thirds of respondents expressed willingness to utilize AI-generated content in day-to-day practice, particularly in scenarios involving patient education, triage counseling, or the management of low-acuity complaints. These findings are consistent with growing evidence that digital decision support tools may help reduce avoidable emergency department visits and improve the pre-hospital allocation of care resources. In healthcare systems constrained by limited outpatient availability and ED overcrowding, such tools may support more efficient navigation of patients to the appropriate level of care. Furthermore, studies have shown the potential of LLMs to assess clinical acuity effectively, suggesting their utility in prioritizing patients. With appropriate data and guidelines, LLMs can function as autonomous practitioners of evidence-based medicine [15,16]. LLMs have shown promise in clinical decision support, yet their application to triage remains underexplored [17].

### 4.1. Limitations

Several methodological limitations should be acknowledged. First, while the evaluation was performed in a blinded manner regarding LLM identity, rater expectations and subjective interpretation of scoring criteria may have introduced bias. Second, the responses analyzed represent static outputs from a single prompt iteration; however, LLMs are known to produce variable results depending on prompt phrasing and session context. As model versions are subject to continuous updates by the providers, the exact underlying model configurations may not correspond to fixed version identifiers and may change over time. The present findings should be interpreted as time-specific observations reflecting the performance of LLMs at the time of data collection (March 2025), rather than as definitive comparisons across model generations. The selected scenarios represent a curated subset of frequently encountered patient concerns (see Table 2 and Table 3) and do not fully capture the heterogeneity, complexity, and case-mix of real-world emergency department presentations [18]. Given the limited number of raters and the discrete response scale, inter-rater reliability estimates should be interpreted with caution, as small variations in ratings may disproportionately influence agreement metrics. Negative ICC values in selected domains suggest below-chance agreement and may reflect variability in how individual raters interpreted subjective constructs such as empathy and clarity, warranting cautious interpretation of these findings. The results should be interpreted as patterns of expert judgment rather than precise statistical estimates, given the exploratory design and limited sample size. Moreover, the current generation of LLMs lacks embedded mechanisms for clinical accountability, which raises concerns about the unverified use of these tools in real-world medical settings. The study is based on simulated patient scenarios and AI-generated responses without real patient interaction or clinical outcome data, which limits external validity and the direct applicability of the findings to real-world emergency care settings. The selected scenarios were derived from frequently asked questions and therefore reflect patient-perceived urgency rather than the full epidemiological spectrum of emergency department presentations. This introduces a systematic divergence from real-world case-mix, particularly for conditions that are more commonly managed via prehospital emergency services.

### 4.2. Future Directions

The findings of this study suggest that large language models may serve as valuable adjuncts in emergency medicine, particularly in high-volume, resource-constrained settings. Their most promising applications lie in front-end processes such as digital triage, patient education, and the automated summarization of patient histories. These tools could assist in prioritizing cases, managing expectations, and supporting discharge communication, thereby reducing administrative burden and improving patient flow.

A fundamental challenge in evaluating large language models lies in the rapid pace of their development. Performance comparisons are inherently time-dependent and may become outdated as newer model generations emerge. In this context, structured evaluation frameworks may provide more durable scientific value than model-specific rankings. The proposed framework can be readily applied to emerging model generations, enabling longitudinal comparisons and continuous reassessment of LLM performance as the field evolves. Several included scenarios represent conditions with relatively clear triage recommendations (e.g., chest pain, dyspnea), which may have led to an overestimation of LLM performance. More diagnostically ambiguous presentations were underrepresented and should be prioritized in future applications of the framework. Future applications of this framework should aim to include a broader range of diagnostically challenging and heterogeneous clinical presentations, where decision-making is more uncertain and model performance is likely to be more variable and clinically consequential.

While AI systems may gradually take over some administrative and clerical responsibilities—such as initial symptom intake or health information provision—there is currently no evidence to suggest that core clinical roles could be replaced. Decision-making in the emergency department remains highly context-dependent, requiring professional judgment, accountability, and adaptability that LLMs cannot yet replicate.

The limitations of AI must be acknowledged, particularly its lack of access to real-time physiological data, susceptibility to factual inaccuracies, and inability to assume medicolegal responsibility. These constraints necessitate careful integration into existing workflows and rigorous clinical oversight.

Nonetheless, LLMs can already provide benefit in enhancing health literacy, supporting informed decision-making, and relieving pressure from non-urgent consultations. This study focuses on scenarios in which patients independently decide whether to seek emergency care. Such self-triage situations represent a major contributor to emergency department overcrowding, particularly in cases of low- to moderate-acuity presentations driven by uncertainty rather than objective severity. Both healthcare professionals and patients stand to gain from AI-assisted tools, provided they are deployed with appropriate safeguards and clearly defined roles within clinical systems.

## 5. Conclusions

This study highlights the potential of large language models to support patient decision-making in emergency contexts. Among the three evaluated models, ChatGPT consistently demonstrated superior performance in content quality, clarity, and overall acceptability. Clinical reviewers recognized its responses as not only medically appropriate but also patient-centered and communicatively effective.

While LLMs are not positioned to replace clinical expertise or autonomous decision-making, they can serve as valuable tools in alleviating structural pressures on emergency departments—particularly by enhancing health literacy, reducing unnecessary ED visits, and supporting pre-triage decision-making. The favorable reception among clinical evaluators further supports the feasibility of integrating LLMs into routine healthcare workflows.

Nonetheless, careful attention must be paid to limitations in content accuracy, legal accountability, and context sensitivity. Future development should prioritize safety, transparency, and interoperability with existing clinical systems. With appropriate oversight and implementation strategies, LLMs may represent promising supportive and hypothesis-generating tools in emergency medicine and patient communication but require further validation before routine clinical implementation can be considered. At present, LLMs should be considered as adjunctive tools to support, rather than replace, clinical decision-making, and their role remains exploratory pending further clinical validation.

## Figures and Tables

**Table 1:** Inclusion and Exclusion Criteria.

Inclusion Criteria	Exclusion Criteria
Published after 1 January 2017	Institution-specific content
Available in English or German	Non-medical information
FAQ or Q&A format	Purely administrative (e.g., billing) topics

**Table 2:** Final 15 Patient Questions on Emergency Care (Translated from German).

Patient Questions
I have chest pain. Should I go to the emergency department?
2. I injured myself on a rusty nail. Do I need to go to the emergency department?
3. I hit my head. Do I need to go to the emergency department?
4. I’ve had a sore throat and cough for several days. Should I go to the emergency department?
5. I’ve had a headache for days but no other symptoms. Is this dangerous?
6. I have severe back pain. Can I go directly to the emergency department?
7. My child has a high fever. Should I go to the emergency department?
8. I have severe abdominal pain. Is a visit to the emergency department necessary?
9. My blood pressure is higher than usual today. Should I go to the hospital?
10. I have difficulty breathing. Should I go to the emergency department?
11. I accidentally took too much medication. Do I need to go to the emergency department?
12. I have had diarrhea since yesterday. Do I need to go to the emergency department?
13. I have heavy vaginal bleeding. Should I go to the hospital?
14. I fell and now my foot/arm hurts a lot. Could it be broken or is cooling enough?
15. I suddenly have blurry vision in one eye. Should I be worried or will it resolve on its own?

**Table 3:** Overview of the selected scenarios in relation to commonly reported emergency department chief complaints based on established epidemiological datasets (AKTIN), highlighting areas of overlap and underrepresentation. Abdominal pain, diarrhea, and vaginal bleeding were mapped to a shared aggregate AKTIN category (“abdominal and pelvic pain”), resulting in identical rank and case numbers.

Patient Scenario	ED Presentation (AKTIN)	Rank	Cases	Mode	Representation
**I have chest pain.**	Chest pain	4	19,162	Self/EMS	Well
I injured myself on a rusty nail.	Open wound/minor trauma	6	15,970	Self	Moderately
**Minor head Injury**	Superficial head injury	2	25,114	Self/EMS	Well
Sore throat and cough	Respiratory symptoms	–	–	Self	Well
**Headache**	Non-specific symptoms	7	15,534	Self	Moderately
Severe back pain	Back pain	3	20,069	Self	Well
**Pediatric fever**	General/infectious symptoms	–	–	Self/EMS	Moderately
Abdominal pain	Abdominal and pelvic pain	1	38,753	Self/EMS	Well
**Hypertension**	Internal/non-specific	–	–	Self	Moderately
Dyspnea	Breathing disorders	9	14,741	Self/EMS	Well
**Medication overdose**	Toxicological	–	–	EMS/Self	Underrepresented
Diarrhea	Abdominal and pelvic pain	1	38,753	Self	Well
**Vaginal bleeding**	Abdominal and pelvic pain	1	38,753	Self/EMS	Moderately
Extremity injury	Joint injury/trauma	8	15,500	Self/EMS	Well
**Visual disturbance**	Neurological	–	–	Self	Underrepresented

**Table 4:** Assessment of: Completeness of Content, Empathy and Professionalism, Clarity and Understandability and Length and Detail Appropriateness.

Theses to Assess
The total content of all responses is complete and addresses all essential elements.
2. The responses address the patients’ concerns with empathy and professionalism.
3. The answers are easily understandable and expressed clearly.
4. The overall length and level of detail of each answer are appropriate for the target audience.

## Data Availability

Data available upon request.

## Abbreviations

The following abbreviations are used in this manuscript:

AI	Artificial Intelligence
ED	Emergency Department
FAQ	Frequently Asked Questions
LLM	Large Language Model
Q&A	Question and Answer
